# The vestibular system is critical for the changes in muscle and bone induced by hypergravity in mice

**DOI:** 10.14814/phy2.12979

**Published:** 2016-10-03

**Authors:** Naoyuki Kawao, Hironobu Morita, Koji Obata, Yukinori Tamura, Katsumi Okumoto, Hiroshi Kaji

**Affiliations:** ^1^ Department of Physiology and Regenerative MedicineKindai University Faculty of MedicineOsakasayamaJapan; ^2^ Department of PhysiologyGifu University Graduate School of MedicineGifuJapan; ^3^ Mouse Epigenetics ProjectISS/Kibo ExperimentJapan Aerospace Exploration AgencyTsukubaJapan; ^4^ Life Science Research InstituteKindai UniversityOsakasayamaJapan

**Keywords:** Bone, gravity, muscle, vestibular system

## Abstract

Gravity changes concurrently affect muscle and bone as well as induce alterations in vestibular signals. However, the role of vestibular signals in the changes in muscle and bone induced by gravity changes remains unknown. We therefore investigated the effects of vestibular lesions (VL) on the changes in muscle and bone induced by 3 *g* hypergravity for 4 weeks in C57BL/6J mice. Quantitative computed tomography analysis revealed that hypergravity increased muscle mass surrounding the tibia and trabecular bone mineral content, adjusting for body weight in mice. Hypergravity did not affect cortical bone and fat masses surrounding the tibia. Vestibular lesions blunted the increases in muscle and bone masses induced by hypergravity. Histological analysis showed that hypergravity elevated the cross‐sectional area of myofiber in the soleus muscle. The mRNA levels of myogenic genes such as MyoD, Myf6, and myogenin in the soleus muscle were elevated in mice exposed to hypergravity. Vestibular lesions attenuated myofiber size and the mRNA levels of myogenic differentiation markers enhanced by hypergravity in the soleus muscle. Propranolol, a *β*‐blocker, antagonized the changes in muscle induced by hypergravity. In conclusion, this study is the first to demonstrate that gravity changes affect muscle and bone through vestibular signals and subsequent sympathetic outflow in mice.

## Introduction

Mechanical stress markedly influences both muscle and bone (Wu et al. 2011). Several studies suggest that low‐magnitude mechanical impacts affect muscle and bone in an anabolic manner (Colnot et al. 2012; Evans et al. 2013). Muscle wasting and osteoporosis are serious problems in conditions of reduced mechanical stress such as immobilization. Long‐term space flight causes muscle atrophy and bone loss in astronauts (Fitts et al. 2010; Orwoll et al. 2013). These findings suggest that gravity changes might exert major effects on musculoskeletal systems.

Muscle mass is regulated by muscle regeneration, muscle protein synthesis, and muscle protein degradation. Growth factors, such as insulin‐like growth factor‐1 (IGF‐1), regulate muscle protein synthesis (Perrini et al. 2010). Muscle protein degradation is facilitated by intracellular proteolytic systems such as ubiquitin–proteasome and autophagy systems (Cohen et al. 2015). On the other hand, bone homeostasis is defined by the balance of bone formation and resorption (Sims and Martin 2014). Runt‐related transcription factor 2 (Runx2) and Osterix are master transcription factors for the differentiation of mesenchymal stem cells into osteoblasts. Osteoblasts express alkaline phosphatase (ALP) and osteocalcin (OCN) to control mineralization for bone formation. Bone resorption is mainly regulated by receptor activator of nuclear factor‐*κ*B ligand (RANKL) and osteoprotegerin (OPG). RANKL is crucial for osteoclast formation and activity, and OPG suppresses bone resorption by binding to RANKL. Osteoporosis is caused by an imbalance between osteoblastic bone formation and osteoclastic bone resorption.

Numerous studies indicate that low gravity induces muscle wasting and osteopenia (Fitts et al. 2010; Orwoll et al. 2013). In astronauts, the effects of microgravity on myofiber atrophy are stronger in the soleus muscle, known as an antigravity muscle, than in the gastrocnemius muscle (Fitts et al. 2010). Muscle protein degradation is facilitated by mechanical unloading (Fitts et al. 2010). Microgravity‐induced bone loss is related to an increase in bone resorption and a decrease in calcium absorption in astronauts (Smith et al. 2005). On the other hand, long‐term hypergravity increases the soleus muscle mass and attenuates bone loss induced by ovariectomy in rodents (Frey et al. 1997; Ikawa et al. 2011). However, the precise mechanisms by which the gravity changes affect muscle and bone remain unknown.

Microgravity influences the control of the cardiovascular system, followed by increased cardiac output and decreased diastolic arterial pressure in astronauts in space (Norsk and Christensen 2009). Moreover, microgravity reduced ocular counter‐rolling and vergence in monkeys (Dai et al. 1994, 1996), suggesting that otolith‐ocular reflexes are decreased when exposed to microgravity. We previously revealed that baroreceptor and vestibulosympathetic reflexes are involved in arterial blood pressure control during hypergravity in rats (Gotoh et al. 2004). The vestibular system has a high level of plasticity, and gravity changes induce plastic alteration of vestibular functions such as vestibulosympathetic and vestibulo‐ocular reflexes (Clarke et al. 2000; Abe et al. 2008). Vestibular system plasticity is thought to be involved in orthostatic intolerance in astronauts after space flight (Yates et al. 2000). These findings suggest that vestibular signals mediate the effects of gravity changes on the cardiovascular system. Previous studies showed that labyrinthectomies and vestibular lesions (VL) affect bone remodeling and reduce bone mineral density (BMD) through the sympathetic nervous system in rodents (Levasseur et al. 2004; Vignaux et al. 2013). Moreover, Luxa et al. (2013) reported that neurochemical vestibular deafferentation induces increased myofiber remodeling and NFATc1‐myonuclear translocation in rat postural skeletal muscle. These findings raise the possibility that the vestibular system might play a role in the alterations in muscle and bone induced by gravity changes.

Muscle sympathetic outflow is moderately increased in astronauts in space (Ertl et al. 2002). Moran et al. (2001) revealed that hypergravity increases the urinary levels of epinephrine and norepinephrine in mice. The sympathetic nervous system regulates muscle mass, and the chronic administration of *β*‐adrenergic receptor agonists increases muscle mass (Lynch and Ryall 2008). On the other hand, propranolol attenuated VL‐ and unloading‐induced bone loss in rodents (Levasseur et al. 2003; Denise et al. 2009; Vignaux et al. 2013). These findings suggest that gravity changes might affect muscle and bone via sympathetic outflow.

In this study, we therefore investigated the role of vestibular signaling and sympathetic outflow on the effects of hypergravity in muscle and bone using vestibular‐lesioned mice and propranolol, a blocker of *β*‐adrenergic receptors.

## Materials and Methods

### Animals

Animals used in this study were maintained in accordance with the “Guiding Principles for Care and Use of Animals in the Field of Physiological Science” set by the Physiological Society of Japan. The experiments were approved by the Animal Research Committees of Gifu University (25‐53) and Japan Aerospace Exploration Agency (014‐007). Male C57BL/6J mice were obtained from Chubu Kagaku Shizai Co., Ltd. (Nagoya, Japan). Six‐week‐old mice (*n *=* *64) were randomly divided into four groups: 1 *g* sham animals (*n* = 16), 3 *g* sham animals (*n* = 16), 1 *g* VL animals (*n* = 17), and 3 *g* VL animals (*n* = 15). Eight mice in each group were randomly selected and used for the real‐time PCR‐based gene expression analysis. The other mice were used for the quantitative computed tomography (QCT) and histological analyses. Eight‐week‐old mice (*n* = 48) were also used to examine the effects of propranolol on the changes in muscle and bone induced by hypergravity. The mice were randomly divided into four groups: 1 *g* mice treated with vehicle (*n* = 12), 3 *g* mice treated with vehicle (*n* = 12), 1 *g* mice treated with propranolol (*n* = 12), and 3 *g* mice treated with propranolol (*n* = 12). Six mice in each group were randomly selected for the real‐time PCR‐based analysis, and the other six mice in each group were used for the QCT and histological analyses. A 3 *g* environment was induced by centrifugation, and the cages (30 cm × 18 cm × 14 cm) were set in a custom‐made gondola‐type rotating box with a 1.5 m arm (Shimadzu, Kyoto, Japan) for 4 weeks as described previously (Abe et al. 2010). To obtain 3 *g* environment, 41 rpm was applied. The centrifuge was stopped daily for 30 min to clean the cages and to supply food and water. Mice with exposure to 1 *g* and 3 *g* were kept in the same experimental room. All the mice accessed food and water ad libitum. The room temperature was maintained at 24 ± 1°C with a 12‐h:12‐h light/dark cycle.

### Bilateral vestibular lesion model

Vestibular lesion (VL) was induced in mice according to the method described previously (Abe et al. 2010; Morita et al. 2015). Briefly, 6‐week‐old mice were anesthetized using 2% isoflurane, and the malleus, anvil, and stapes were removed through the outer ear. The vestibule was lesioned using a dental reamer (size: 20, MANI, Inc., Utsunomiya, Japan) through an oval ear window, and a high‐frequency current was applied to the vestibule via the reamer using a cautery apparatus (Solid State Electrosurgery, MS‐1500, MERA, Senko Medical Instrument Manufacturing Co., Ltd., Tokyo, Japan). The parameters of cautery technique were with the aim of inducing vestibular hair cell lesions and destroying the vestibular epithelium in histological sections of the cochleo‐vestibular organ. The effectiveness of the VL procedure was checked by swimming test, as described previously (Abe et al. 2010; Morita et al. 2015), in which mice with complete VL continued to turn around under warm water, but mice with incomplete VL did not. The surgery was performed bilaterally at the same time. For the sham VL surgery, the tympanic membrane was removed, but the auditory ossicles were left intact. Penicillin G potassium (3000 U/kg, Meiji Seika Pharma, Tokyo, Japan) and buprenorphine (3 *μ*g/kg, Lepetan, Otsuka, Tokyo, Japan) were administered subcutaneously. After the mice had been allowed to recover from the VL‐inducing surgery for 14 days, they were transferred to the cages on the centrifuge and then kept in the 3 *g* or 1 *g* environment for 4 weeks. Next, samples were collected for the analyses as described below. Although the food and water intake of the mice fell after the VL‐inducing surgery, they were restored to basal levels by day 14. We therefore started the experiments on 14 days after the surgery.

### Drug administration

Propranolol (Sigma, St. Louis, MO) was dissolved in physiological saline and administered to 8‐week‐old mice at intraperitoneal doses of 10 mg/kg for the initial 5 days of the experiments. Propranolol was administered via drinking water at a concentration of 0.5 g/L from day 5 to day 28.

### Quantitative computed tomography analysis

Quantitative computed tomography (QCT) analysis was performed according to our previous study (Tamura et al. 2014) and the guidelines of the American Society for Bone and Mineral Research (Bouxsein et al. 2010). Briefly, after the mice were euthanized with excess isoflurane, hind limbs of mice were scanned using an X‐ray CT system in vivo (Latheta LCT‐200; Hitachi Aloka Medical, Tokyo, Japan). CT images were acquired using the following parameters: 50 kVp tube voltage, 500 *μ*A tube current, 3.6 msec integration time, 48 mm axial field of view, and 24 *μ*m isotropic voxel size for the analysis of the tibia and 48 × 48 × 192 *μ*m voxel size for the analysis of muscle and fat tissues surrounding the tibia. Regions of interest (ROIs) were defined between the ends of the proximal and distal tibia for the assessment of muscle and fat mass surrounding the tibia. Fat mass was expressed as total mass of fat tissue surrounding the tibia including subcutaneous and intramuscular fat. ROIs were defined as 1680 *μ*m (70 slices) segments from 96 *μ*m distal to the end of the proximal growth plate toward the diaphysis (proximal tibial metaphysis) for assessment of total BMD, total bone mineral content (BMC), trabecular BMD, and trabecular BMC. For the assessment of cortical BMD, cortical BMC, cortical thickness, and cortical area, ROIs were defined as 2160 *μ*m (90 slices) segments of the mid‐diaphysis of the tibia. These bone parameters were analyzed using LaTheta software (version 3.40). A threshold density of 160 mg/cm was selected to distinguish mineralized from unmineralized tissue. The density range was calibrated daily with a manufacturer‐supplied phantom. The coefficient of variation for bone parameters obtained from 10 repeated measurements with a bone sample was less than 0.2%.

### Histological analysis

Under isoflurane anesthesia, the mice were perfused transcardially with physiological saline and subsequently with 4% paraformaldehyde in a phosphate buffer (pH 7.4). The soleus, gastrocnemius, and quadriceps muscles were removed and embedded in paraffin. Four‐micrometer‐thick sections were obtained and stained with hematoxylin/eosin. The hematoxylin/eosin‐stained sections were photographed under a microscope (E800; Canon, Tokyo, Japan) with a CCD camera and cross‐sectional areas of, at least, 500 myofibers were quantified by using Mac SCOPE (Mitani Co., Fukui, Japan) in a blinded manner. Immunostaining was performed as described previously (Kawao et al. 2013, 2014). Briefly, the sections were incubated with rabbit polyclonal anti‐Pax7 antibody (# LS‐B9285; LifeSpan BioSciences, Inc., Seattle, WA) at a dilution of 1:300 and rat monoclonal anti‐laminin antibody (# LS‐C152900; LifeSpan BioSciences, Inc.) at a dilution of 1:100, followed by incubation with the appropriate secondary antibody. A tyramide signal amplification system (PerkinElmer, Waltham, MS) was used to detect immunopositive signals. These sections were counterstained with diamino phenylindole. The numbers of Pax7‐positive cells per 0.1 mm^2^ of the microscopic fields were quantified. Five sections obtained from each muscle were used for the quantitative analysis.

### Quantitative real‐time PCR

Total RNA was isolated from the tissues using an RNeasy Mini Kit (Qiagen, Hilden, Germany). The incorporation of SYBR Green into double‐stranded DNA was assessed by quantitative real‐time PCR using an ABI StepOne Real‐Time PCR System (Applied Biosystems, Carlsbad, CA) as described previously (Kawao et al. 2013). The primers for real‐time PCR are shown in Table 1. The mRNA levels of the target genes were normalized with the glyceraldehyde‐3‐phosphate dehydrogenase (GAPDH) mRNA levels. The amounts of GAPDH mRNA in each group were same in the experiment, since the levels of GAPDH mRNA were similar in all experiments.

**Table 1:** Primers used for real‐time PCR experiments

Gene	Primer sequence
Pax7	
Forward	5′‐CAGTGTGCCATCTACCCATGCTTA‐3′
Reverse	5′‐GGTGCTTGGTTCAAATTGAGCC‐3′
MyoD	
Forward	5′‐AGCACTACAGTGGCGACTCAG‐3′
Reverse	5′‐AGGCGGTGTCGTAGCCATTC‐3′
Myf6	
Forward	5′‐ATGGTACCCTATCCCCTTGC‐3′
Reverse	5′‐TAGCTGCTTTCCGACGATCT‐3′
Myogenin	
Forward	5′‐GCTGCCTAAAGTGGAGATCCT‐3′
Reverse	5′‐GCGCTGTGGGAGTTGCAT‐3′
MHC I	
Forward	5′‐GCCAACTATGCTGGAGCTGATGCCC‐3′
Reverse	5′‐GGTGCGTGGAGCGCAAGTTTGTCATAAG‐3′
MCK	
Forward	5′‐GGCAACACCCACAACAAGTTC‐3′
Reverse	5′‐CCTTGAAGACCGTGTAGGACT‐3′
Atrogin‐1	
Forward	5′‐GTCGCAGCCAAGAAGAGAAAGA‐3′
Reverse	5′‐TGCTATCAGCTCCAACAGCCTT‐3′
MuRF1	
Forward	5′‐TAACTGCATCTCCATGCTGGTG‐3′
Reverse	5′‐TGGCGTAGAGGGTGTCAAACTT‐3′
Beclin1	
Forward	5′‐TGAAATCAATGCTGCCTGGG‐3′
Reverse	5′‐CCAGAACAGTATAACGGCAACTCC‐3′
LC3b	
Forward	5′‐CTGGTGAATGGGCACAGCATG‐3′
Reverse	5′‐CGTCCGCTGGTAACATCCCTT‐3′
Runx2	
Forward	5′‐AAATGCCTCCGCTGTTATGAA‐3′
Reverse	5′‐GCTCCGGCCCACAAATCT‐3′
Osterix	
Forward	5′‐AGCGACCACTTGAGCAAACAT‐3′
Reverse	5′‐GCGGCTGATTGGCTTCTTCT‐3′
ALP	
Forward	5′‐ATCTTTGGTCTGGCTCCCATG‐3′
Reverse	5′‐TTTCCCGTTCACCGTCCAC‐3′
Col I	
Forward	5′‐GGTCAAAGGTTTGGAAGCAG‐3′
Reverse	5′‐TGTGAAATGCCACCTTTTGA‐3′
Osteocalcin	
Forward	5′‐CCTGAGTCTGACAAAGCCTTCA‐3′
Reverse	5′‐GCCGGAGTCTGTTCACTACCTT‐3′
RANKL	
Forward	5′‐ CACAGCGCTTCTCAGGAGCT‐3′
Reverse	5′‐CATCCAACCATGAGCCTTCC‐3′
OPG	
Forward	5′‐AGTCCGTGAAGCAGGAGT‐3′
Reverse	5′‐CCATCTGGACATTTTTTGCAAA‐3′
GAPDH	
Forward	5′‐AGGTCGGTGTGAACGGATTTG‐3′
Reverse	5′‐GGGGTCGTTGATGGCAACA‐3′

MHC I, myosin heavy chain I; MCK, muscle creatine kinase; ALP, alkaline phosphatase; Col I, type I collagen; RANKL, receptor activator of nuclear factor *κ*B ligand; OPG, osteoprotegerin; GAPDH, glyceraldehyde‐3‐phosphate dehydrogenase.

### Blood chemistry

Serum corticosterone levels were determined using OCTAEIA Corticosterone enzyme immunoassay kit (Immunodiagnostic systems, Fountain Hills, AZ). Serum bone‐specific alkaline phosphatase (BAP) and cross‐linked C‐telopeptide of type I collagen (CTX) were measured using a Mouse Bone‐specific Alkaline Phosphatase ELISA kit (My BioSource, San Diego, CA) and a RatLaps EIA kit (Immunodiagnostic Systems, Fountain Hills, AZ), respectively.

### Statistical analysis

Data are represented as the mean ± standard error of the mean (SEM). Two‐way analysis of variance followed by Tukey–Kramer test was performed for multiple comparisons. Differences among the experimental groups were considered significant when *P* values were less than 0.05.

## Results

### Effects of VL on hypergravity‐induced changes in body weight and muscle and fat masses surrounding the tibia in mice

**Figure 1:**
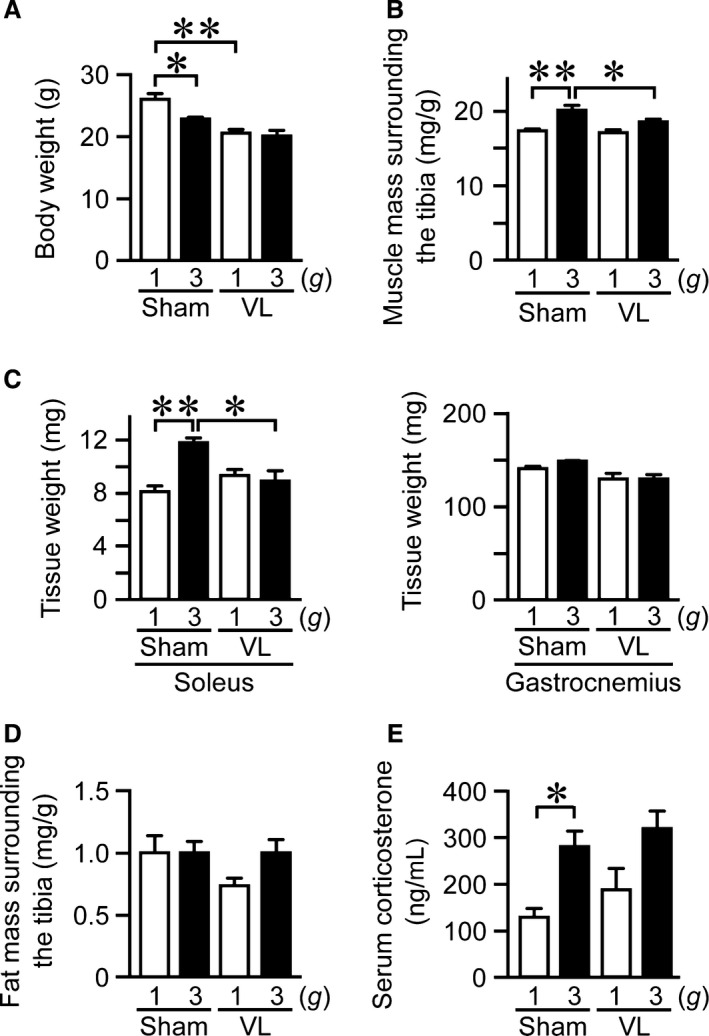
Effects of vestibular lesion (VL) on hypergravity‐induced changes in body weight, muscle, and fat masses. Body weight (A), muscle mass surrounding the tibia (B), tissue weight of soleus and gastrocnemius muscles (C), and fat mass surrounding the tibia (D) in mice with VL or sham operation after exposure to 1 *g* or 3 *g* for 4 weeks. Muscle mass (B) and fat mass (D) surrounding the tibia were assessed by QCT and adjusted for body weight. (E) Serum sample was collected from mice with sham or VL surgery after exposure to 1 *g* or 3 *g* for 4 weeks. Then, serum corticosterone levels were quantified as described in Materials and Methods. Data represent the mean ± SEM of 8 (1 *g* sham and 3 *g* sham groups), 9 (1 *g* VL group), and 7 (3 *g* VL group) mice (A, B, D). Data represent the mean ± SEM of 4 (C) and 8 (E) mice in each group. *P < 0.05. **P < 0.01.

Both 3 *g* or VL mice experienced significantly reduced body weight compared to control group (Fig. 1A). Since body weight loss modulates muscle and fat mass changes, we evaluated the muscle and fat masses surrounding the tibia by adjusting for body weight. The muscle mass surrounding the tibia was increased in 3 *g* mice compared to 1 *g* mice (Fig. 1B), although the fat masses surrounding the tibia were similar between 1 *g* and 3 *g* mice (Fig. 1D). Vestibular lesions significantly attenuated the increase in muscle mass surrounding the tibia induced by hypergravity (Fig. 1B). Moreover, VL blunted tissue weight elevated by hypergravity in the soleus muscle in mice (Fig. 1C). Hypergravity significantly elevated serum corticosterone levels in mice, although VL did not affect them (Fig. 1E).

### Effects of VL on hypergravity‐induced changes in tibial bone mass in mice

**Figure 2:**
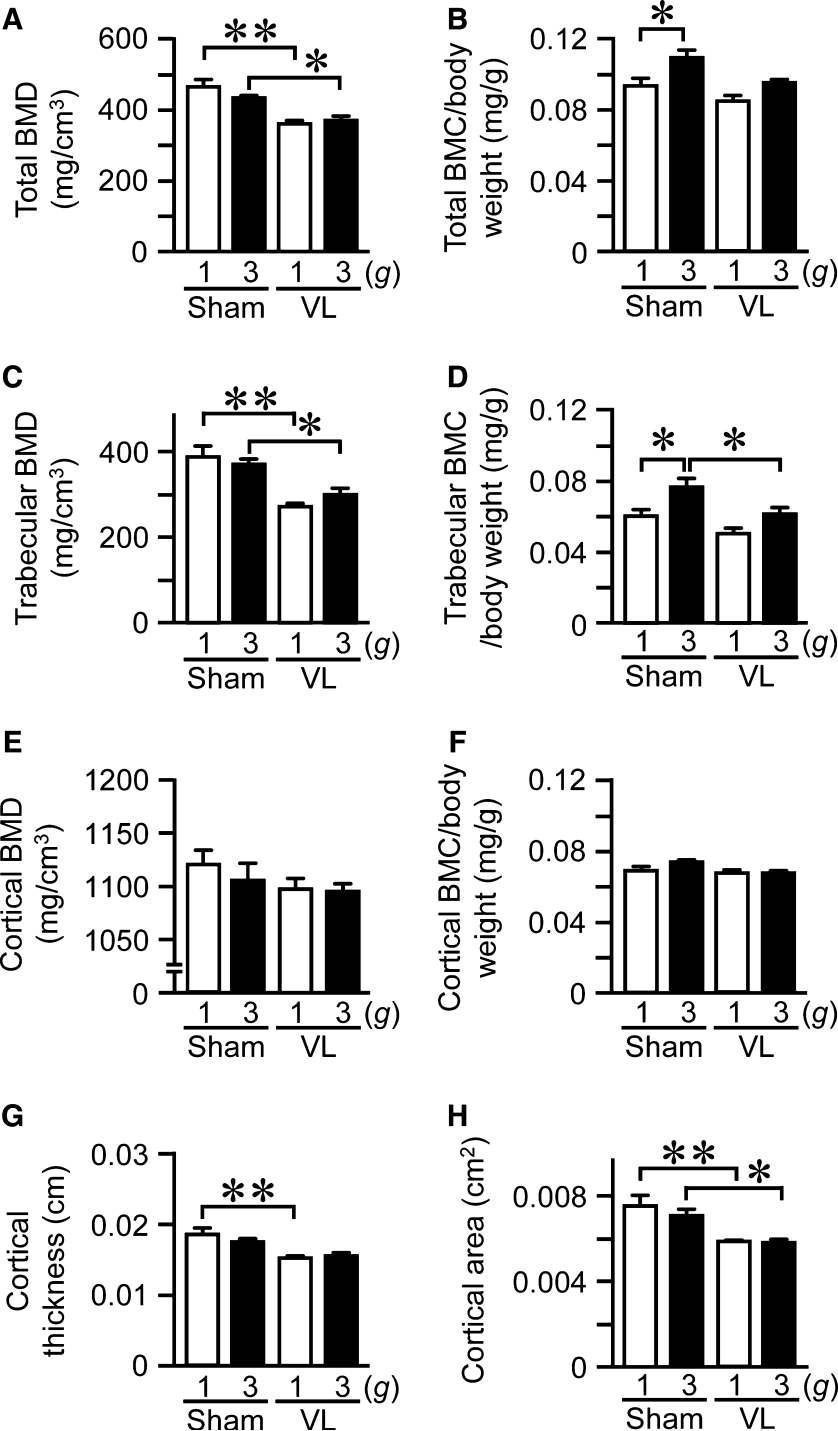
Effects of vestibular lesion (VL) on hypergravity‐induced changes in bone. Total bone mineral density (BMD) (A), total bone mineral content (BMC) (B), trabecular BMD (C), trabecular BMC (D), cortical BMD (E), cortical BMC (F), cortical thickness (G), and cortical area (H) in tibias of mice with VL or sham operation after exposure to 1 *g* or 3 *g* for 4 weeks were assessed by quantitative computed tomography (QCT). Total BMC (B), trabecular BMC (D), and cortical BMC (F) were adjusted for body weight. Data represent the mean ± SEM of 8 (1 *g* sham and 3 *g* sham groups), 9 (1 *g* VL group), and 7 (3 *g* VL group) mice (A–H). *P < 0.05. **P < 0.01.

Bone mass was evaluated by adjusting for body weight. Total and trabecular BMC were significantly elevated in 3 *g* mice compared to 1 *g* mice (Fig. 2B, D), although hypergravity did not affect total and trabecular BMD in the tibia (Fig. 2A, C). VL significantly blunted the increase in trabecular BMC by hypergravity (Fig. 2D). VL itself significantly reduced the total and trabecular BMD in the tibia (Fig. 2A, C), compatible with previous reports (Levasseur et al. 2004; Vignaux et al. 2013). Hypergravity did not affect cortical BMD, cortical BMC, cortical thickness, or cortical area (Fig. 2E–H), although VL significantly decreased cortical thickness and cortical area (Fig. 2G, H).

### Effects of VL on hypergravity‐induced changes in myofiber size and muscle differentiation in mice

**Figure 3:**
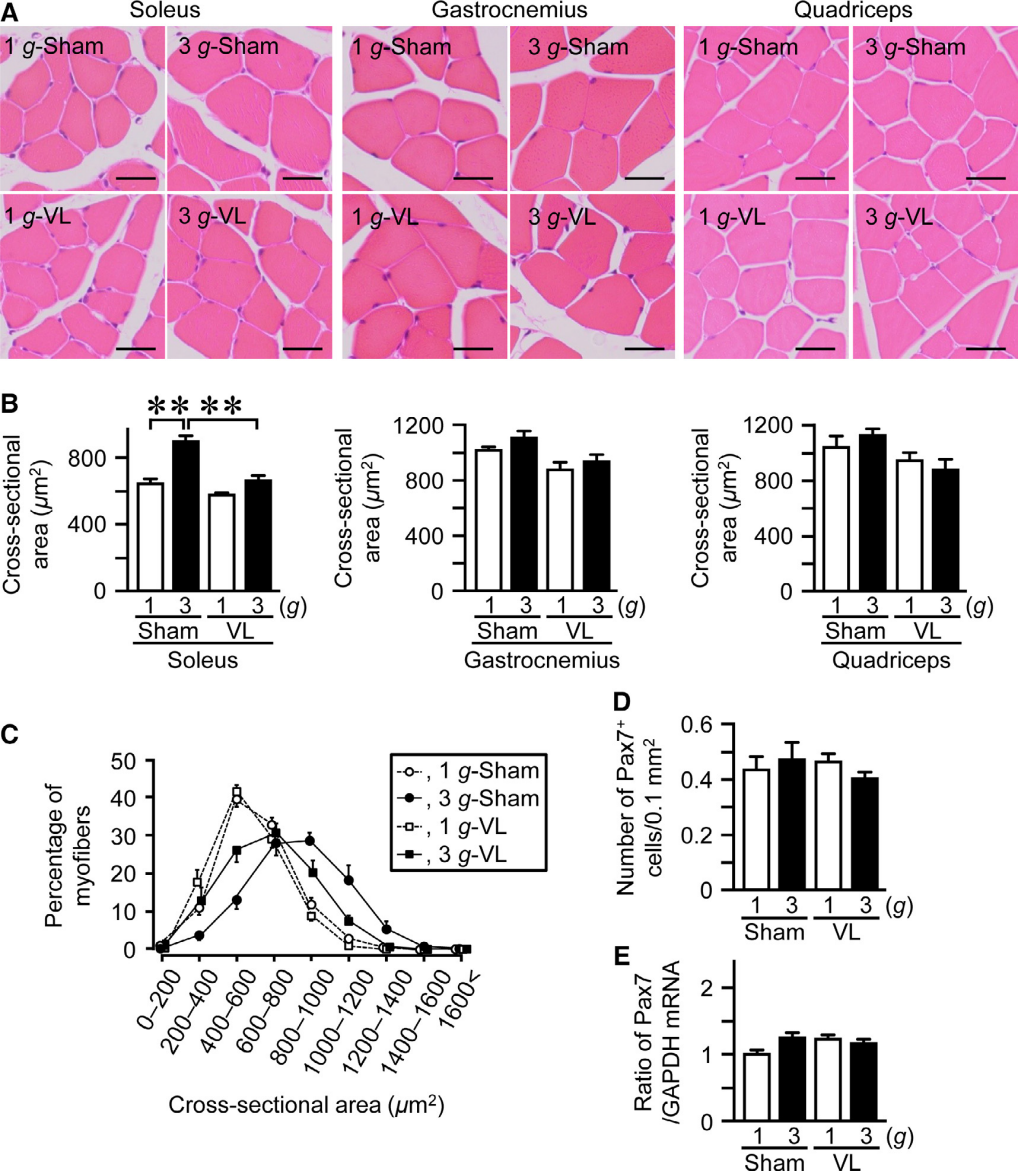
Effects of vestibular lesion (VL) on hypergravity‐induced changes in myofiber in the muscles. Hematoxylin and eosin‐stained sections (A) and cross‐sectional area (B) of myofiber in the soleus, gastrocnemius, and quadriceps muscles of mice with VL or sham operation after exposure to 1 *g* or 3 *g* for 4 weeks. Scale bars indicate 25 *μ*m (A). (C) Distribution of myofiber size of the soleus muscle in mice with VL or sham operation after exposure to 1 *g* or 3 *g* for 4 weeks. Number of Pax7‐positive (Pax7^+^) cells assessed by immunohistochemistry (D) and the levels of Pax7 mRNA assessed by real‐time PCR (E) in the soleus muscles of mice with VL or sham operation after exposure to 1 *g* or 3 *g* for 4 weeks. Data represent the mean ± SEM of 8 (1 *g* sham and 3 *g* sham groups), 9 (1 *g* VL group), and 7 (3 *g* VL group) mice (B–D). Data represent the mean ± SEM of eight mice in each group (E). **P < 0.01.

We next examined the effects of VL on the hypergravity‐induced changes in myofiber size in mice. The cross‐sectional area of myofiber in the soleus muscle was elevated in 3 *g* mice compared to 1 *g* mice (Fig. 3A–C). VL blunted the hypergravity‐induced increases in the cross‐sectional area of myofiber in the soleus muscle (Fig. 3A–C). Neither hypergravity nor VL affected the cross‐sectional area of myofiber in the gastrocnemius or quadriceps muscles (Fig. 3A, B).

Muscle satellite cells play a significant role in the development and regeneration of skeletal muscle (Relaix and Zammit 2012). We next examined the number of Pax7‐positive cells (a marker for muscle satellite cells) and the levels of Pax7 mRNA in the soleus muscle to investigate the involvement of muscle satellite cells in the effects of hypergravity. Neither hypergravity nor VL affected the number of Pax7‐positive cells or Pax7 mRNA levels in the soleus muscle (Fig. 3D, E).

**Figure 4:**
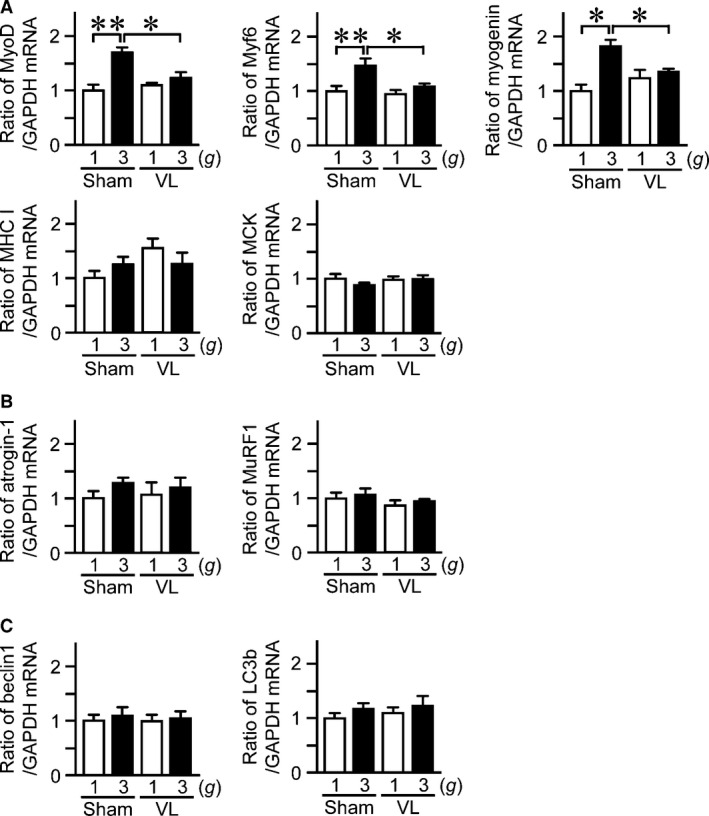
Effects of VL on hypergravity‐induced changes in the mRNA levels of myogenic and protein degradation‐related genes in the soleus muscle. Real‐time PCR analysis of MyoD, Myf6, myogenin, myosin heavy chain I (MHC I), muscle creatine kinase (MCK) (A), atrogin‐1, MuRF1 (B), beclin1, and LC3b (C) in the soleus muscles of mice with VL or sham operation after exposure to 1 *g* or 3 *g* for 4 weeks. Data represent the mean ± SEM of eight mice in each group (A–C). *P < 0.05. **P < 0.01.

We furthermore examined the changes in the mRNA levels of myogenic genes in the soleus muscle following hypergravity and VL. The mRNA levels of MyoD, Myf6, and myogenin in the soleus muscle were elevated in 3 *g* mice compared to 1 *g* mice, but myosin heavy chain I (MHC I) and muscle creatine kinase (MCK) were not elevated (Fig. 4A). VL attenuated the increases in mRNA levels of MyoD, Myf6, and myogenin induced by hypergravity (Fig. 4A). Ubiquitin and autophagy pathways negatively regulate muscle mass (Cohen et al. 2015). We therefore examined the mRNA levels of atrogin‐1 and MuRF1, ubiquitin E3 ligases, as well as beclin1 and LC3b, components of autophagy in muscle cells. Neither hypergravity nor VL affected the mRNA levels of atrogin‐1 or MuRF1 as well as beclin1 or LC3b in the soleus muscle (Fig. 4B, C).

### Effects of hypergravity and VL on bone metabolism in mice

**Figure 5:**
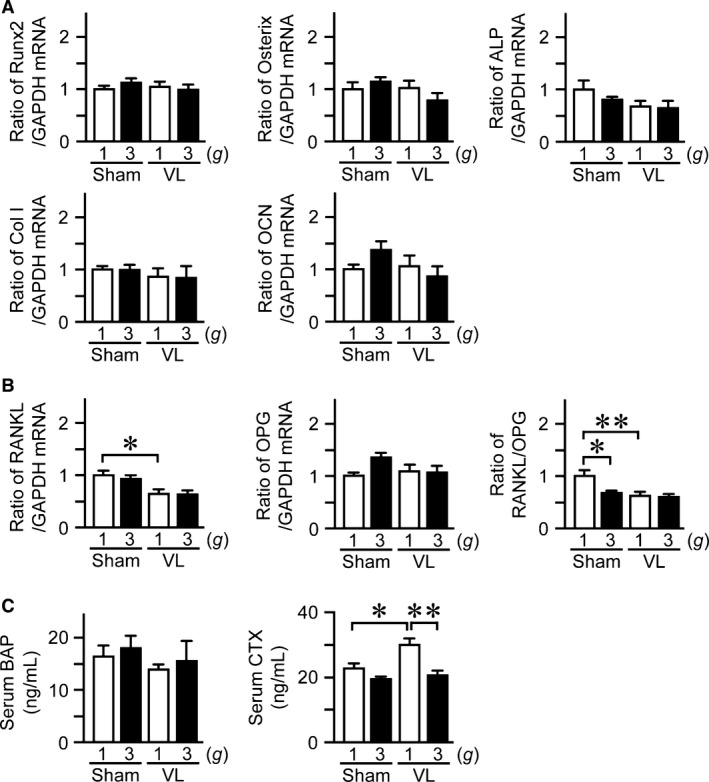
Effects of hypergravity and VL on bone metabolism. Real‐time PCR analysis of Runx2, Osterix, ALP, type I collagen (Col I), OCN (A), RANKL, OPG, and the ratio of RANKL/OPG (B) in the tibias of mice with VL or sham operation after exposure to 1 *g* or 3 *g* for 4 weeks. (C) Serum levels of BAP and CTX in mice with VL or sham operation after exposure to 1 *g* or 3 *g* for 4 weeks. Data represent the mean ± SEM of eight mice in each group (A–C). *P < 0.05. **P < 0.01.

We studied the effects of hypergravity and VL on the mRNA levels of osteogenic genes, such as Runx2, Osterix, ALP, type I collagen, and OCN. Hypergravity and VL did not affect the mRNA levels of Runx2, Osterix, ALP, type I collagen, or OCN in the tibia (Fig. 5A). We next examined the mRNA levels of RANKL, a osteoclast differentiation factor, and OPG, a decoy receptor for RANKL, in the tibia. Although VL reduced the mRNA levels of RANKL, hypergravity did not affect the levels of RANKL or OPG mRNA (Fig. 5B). Moreover, hypergravity or VL significantly decreased the ratio of RANKL/OPG in the tibia, although the effects of both hypergravity and VL were similar to the effects of only hypergravity (Fig. 5B). Third, we examined the effects of hypergravity and VL on serum bone metabolic indices in mice. Hypergravity did not affect serum levels of BAP, a bone formation marker, or CTX, a bone resorption marker, in mice (Fig. 5C). Although VL itself increased serum levels of CTX, hypergravity significantly blunted these VL‐induced elevations of CTX in mice (Fig. 5C).

### Effects of propranolol on the changes in muscle and bone induced by hypergravity in mice

**Figure 6:**
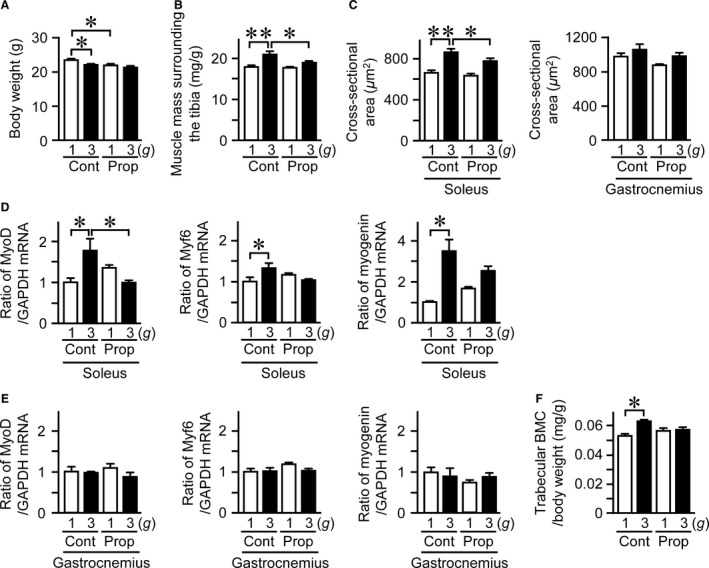
Effects of propranolol on hypergravity‐induced changes in muscle and bone. Body weight (A) and muscle mass surrounding the tibia (B) in mice treated with vehicle (Cont) or propranolol (Prop) after exposure to 1 *g* or 3 *g* for 4 weeks. Muscle mass surrounding the tibia was assessed by quantitative computed tomography (QCT) and adjusted for body weight (B). (C) Cross‐sectional area of myofiber in the soleus and gastrocnemius muscles of mice treated with vehicle or propranolol after exposure to 1 *g* or 3 *g* for 4 weeks. Real‐time PCR analysis of MyoD, Myf6, and myogenin in the soleus (D) and gastrocnemius (E) muscles of the mice. (F) Trabecular bone mineral content (BMC) adjusted for body weight in the tibia of the mice was assessed by QCT. Data represent the mean ± SEM of six mice in each group (A–F). *P < 0.05. **P < 0.01.

Gravity changes affect sympathetic outflow in rodents, and vestibular system is physiologically linked to the sympathetic nerve system (Gotoh et al. 2004). We therefore examined the effects of propranolol on the hypergravity‐induced changes in muscle and bone. Body weight was decreased in mice treated with propranolol compared to that in control mice (Fig. 6A). Propranolol significantly attenuated the increase in muscle mass surrounding the tibia adjusting for body weight induced by hypergravity in mice (Fig. 6B). Moreover, propranolol significantly blunted the increases in cross‐sectional area of myofiber and the mRNA levels of MyoD induced by hypergravity in the soleus muscle (Fig. 6C, D). Propranolol did not affect the cross‐sectional area and mRNA levels of MyoD, Myf6, and myogenin in the gastrocnemius muscle of mice exposed to 1 *g* and 3 *g* environment (Fig. 6C, E). As for bone, propranolol seemed to antagonize the increases in trabecular BMC, adjusting for body weight, induced by hypergravity with no statistical significance (Fig. 6F).

## Discussion

In this study, exposing mice to hypergravity caused their body weight to fall. Our previous study showed that hypergravity decreases food intake, leading to a reduction in body weight in rats (Abe et al. 2010). Although the reduction in food intake induced by hypergravity was gradually ameliorated, the body weight of the rats subjected to hypergravity was still lower than that of the control rats at day 14 (Abe et al. 2010). This observation was similar with the mice subjected to 3 *g* or simulated microgravity during 3 weeks (Gueguinou et al. 2012; Gaignier et al. 2014; Lescale et al. 2015). Numerous studies indicate that body weight and body mass index are positively related to muscle, fat, and bone masses (Rosen and Bouxsein 2006; Muller et al. 2012; Ribeiro and Kehayias 2014). We considered that the body weight reductions induced by hypergravity in the present study might conceal the changes in muscle, bone, and fat mass. We therefore employed body weight‐adjusted muscle, bone, and fat mass values to evaluate the effects of hypergravity on body composition.

This study revealed that hypergravity increases muscle mass adjusting for body weight, myofiber size, and muscle differentiation in the soleus muscles of mice, which is compatible with previous studies (Frey et al. 1997; Fitts et al. 2010). Muscle satellite cells play a primary role during the development and regeneration of muscle in which de novo synthesis of muscle fibers is required (Relaix and Zammit 2012). On the other hand, Lee et al. reported that muscle satellite cells play little role in muscle hypertrophy induced by inhibition of myostatin signaling in mice (Lee et al. 2012). In this study, hypergravity enhanced the mRNA levels of MyoD, Myf6, and myogenin in the soleus muscles of mice, although it did not affect the number of Pax7‐positive cells or the mRNA levels of Pax7, MHC I, MCK, atrogin‐1, MuRF1, beclin1, or LC3b. These results suggest that hypergravity induces muscle hypertrophy in the soleus muscle mainly through the enhancement of myogenic differentiation of myofibers at the differentiation stage other than the stage of satellite cells and terminal stage, but not through muscle protein degradation or autophagy‐related mechanism.

Microgravity induces an increase in bone resorption, associated by severe bone loss in astronauts (Orwoll et al. 2013). Bone loss in astronauts is partly explained by the effects of gravity changes on bone cells. In this study, hypergravity increased bone mass of the trabecular bone adjusting for body weight in mice. Moreover, hypergravity decreased the ratio of RANKL/OPG in the tibias of mice and seemed to suppress serum levels of bone resorption marker elevated by VL, although it did not affect the levels of osteoblast differentiation genes. These findings suggest that hypergravity elevates bone mass adjusting for body weight presumably through the suppression of bone resorption, which is compatible with the evidence that bone resorption is increased in space flight with microgravity (Smith et al. 2005). In our study, hypergravity enhanced body weight‐adjusted trabecular BMC through the vestibular system, but not cortical BMC in mice, although VL reduced cortical thickness and area. Its reason remains unknown at the present time. The effects of hypergravity on cortical bone resorption might counteract the catabolic effects of VL on cortical bone, because hypergravity significantly antagonized bone resorption marker level enhanced by VL in mice.

In this study, VL attenuated the hypergravity‐induced increases in muscle and bone masses adjusting for body weight in mice. Moreover, VL blunted the increases in myofiber size and muscle differentiation of the soleus muscle in mice induced by hypergravity. These findings indicate that vestibular systems are involved in muscle and bone changes induced by hypergravity in mice. Our study revealed that propranolol attenuated the increases in muscle mass adjusting for body weight, myofiber size, and the mRNA levels of myogenic genes induced by hypergravity in mice. We previously showed that the vestibular afferents played a major role in controlling sympathetic nerve activity and then arterial blood pressure during gravity changes (Gotoh et al. 2004). From these evidences, we can speculate that gravity changes might affect the sympathetic outflow through vestibular signals. Since this study showed that hypergravity influences the soleus muscle and trabecular bone masses adjusting for body weight through vestibular signals in mice, gravity changes might affect muscle and bone through vestibular signals and the subsequent sympathetic outflow in mice.

The vestibular system plays a crucial role in the sense of equilibrium and the response to liner and angular gravity acceleration. In addition, the vestibule–spinal reflex plays an important role in posture control by controlling the appendicular muscles, such as the soleus muscle. As we recently reported, the trunk became flat and the tail went up at the onset of 2 *g* environment in mice, although these postural changes were not observed in VL mice during centrifugal acceleration or exposure to 2 *g* (Morita et al. 2015). In our preliminary study, the trunk of mice became flat immediately after 3 *g* centrifugation in mice with sham surgery, and the mice kept the flat posture for 4–7 h. On the other hand, the posture did not change during 3 *g* centrifugation in mice with VL. These findings suggest that the vestibule–spinal reflex controls posture during exposure to hypergravity. We therefore speculated that the vestibulospinal reflex might partly contribute to the changes in muscle and bone induced by hypergravity, although the contribution of the vestibule–spinal reflex to the findings of this study remain unclear due to the methodological difficulties associated with the destruction of the vestibule–spinal tract at our laboratory.

Gravity change is a well‐known stress factor during spaceflight. Gueguinou et al. (2012) revealed that serum corticosterone levels were elevated in mice with exposure to 3 *g* for 21 days, suggesting that chronic exposure to hypergravity induces stress responses in mice. In this study, hypergravity significantly elevated plasma corticosterone levels in mice. These glucocorticoid responses to hypergravity were similar with the results in a study by Gueguinou et al. (2012). However, it does not seem to be probable that glucocorticoid response to stress is responsible for the changes in muscle and bone induced by hypergravity in mice, since glucocorticoid negatively affects muscle and bone masses (Schakman et al. 2009; Tamura et al. 2015).

Appropriate gravity level is a controversial issue to investigate the effects of hypergravity in muscle and bone (Frey et al. 1997; Ikawa et al. 2011; Gueguinou et al. 2012; Bojados and Jamon 2014; Canciani et al. 2015; Gnyubkin et al. 2015). Gnyubkin et al. (2015) revealed that the effects of chronic hypergravity on bone were dependent on the levels of gravity with a threshold between 2 *g* (increased bone mass) and 3 *g* (deleterious for bone). On the other hand, Bojados and Jamon revealed that chronic hypergravity exerts a positive impact on muscle force until 3 *g* in mice (Bojados and Jamon 2014). Moreover, exposure to 3 *g* increased the soleus muscle mass in mice and attenuated the ovariectomy‐induced bone loss in rats (Frey et al. 1997; Ikawa et al. 2011). The present findings that 3 *g* environment for 4 weeks increased muscle and bone masses in mice were compatible with those findings. Moreover, in our preliminary study, the effects of 2 *g* environment on muscle and bone masses seemed to be much less compared to those of 3 *g* environment in mice. Further studies will be necessary to clarify the threshold of the gravity levels in muscle and bone responses during long‐term hypergravity.

## Perspectives and Significance

In conclusion, we demonstrated for the first time that hypergravity affects muscle and bone through vestibular signaling and subsequent sympathetic outflow in mice. Further studies will be necessary to clarify the detailed mechanisms by which hypergravity affect muscle and bone through the vestibular system. The control of vestibular signals might be an effective approach for the management of muscle wasting and osteopenia in space flight and immobilization. Numerous researchers have recently noted interactions between several organs, such as muscle–bone relationships and bone–vascular interactions. Among them, the link between the nervous system and the musculoskeletal system seems to be very important for the understanding of bone metabolism. Recent studies suggest that autonomic, serotonergic, and sensory nervous systems regulate bone remodeling (Karsenty and Oury 2010; Fukuda et al. 2013; Houweling et al. 2015). This study is the first to raise the prospect that the vestibular system is related to the effects of gravity changes in muscle and bone concurrently.

## Conflict of Interest

None declared.
